# COVID-19 Public Opinion: A Twitter Healthcare Data Processing Using Machine Learning Methodologies

**DOI:** 10.3390/ijerph20010432

**Published:** 2022-12-27

**Authors:** Shweta Agrawal, Sanjiv Kumar Jain, Shruti Sharma, Ajay Khatri

**Affiliations:** 1 Institute of Advanced Computing, SAGE University, Indore 452010, India; 2 Electrical Engineering Department, Medi-Caps University, Indore 453331, India; 3 Department of Computer Science and Engineering, Indore Institute of Science &Technology, Indore 453332, India; 4 Bellurbis Technologies Private Limited, Indore 452001, India

**Keywords:** machine learning, polarity, sentiment analysis, classification, accuracy, healthcare, social media, artificial intelligence

## Abstract

The COVID-19 pandemic has shattered the whole world, and due to this, millions of people have posted their sentiments toward the pandemic on different social media platforms. This resulted in a huge information flow on social media and attracted many research studies aimed at extracting useful information to understand the sentiments. This paper analyses data imported from the Twitter API for the healthcare sector, emphasizing sub-domains, such as vaccines, post-COVID-19 health issues and healthcare service providers. The main objective of this research is to analyze machine learning models for classifying the sentiments of people and analyzing the direction of polarity by considering the views of the majority of people. The inferences drawn from this analysis may be useful for concerned authorities as they work to make appropriate policy decisions and strategic decisions. Various machine learning models were developed to extract the actual emotions, and results show that the support vector machine model outperforms with an average accuracy of 82.67% compared with the logistic regression, random forest, multinomial naïve Bayes and long short-term memory models, which present 78%, 77%, 68.67% and 75% accuracy, respectively.

## 1. Introduction

The COVID-19 outbreak has severely affected countries all over the globe since December 2019. It had a tremendous impact on both society and the economy [1,2]. The COVID-19 epidemic became the main topic of news and research and gained a lot of attention from national and international media and researchers. A previous study on pandemic communication found that the content covered by the news media has a strong influence on how people seek information, evaluate it and make concerned decisions [3,4]. Indeed, in crises such as public health threats, news coverage is widely believed to have a significant impact on people’s perceptions and behavior [5]. Since the advent of COVID-19, the number of postings about the disease has increased at a highly accelerated rate on Twitter, a popular social media platform for spreading and exchanging information. These tweets include posts by civilians and news agencies through their official Twitter accounts. Social media plays a key role in developing interest in particular topics, which could assist in addressing public concerns, increasing public satisfaction and facilitating the government’s implementation of COVID-19 prevention strategies. Based on the agenda-setting hypothesis, there is a significant link between media coverage of specific problems and public perception [6]. Analysis of social media data helps in planning and understanding public sentiment. Machine learning classifiers, natural language processing, ensemble learning and sentiment analysis play a vital role in helping with this analysis and in useful information extraction [7]. However, it is very difficult to determine whether various social media platforms provide vital information. This is due to several reasons, such as spelling mistakes, the use of abbreviations and semantic obscurity.

Sentiment analysis can be used to analyze data from social media sites such as blogs, wikis, micro-blogging and other online platforms [8]. It is a type of emotional computing that classifies the text into positive, negative and neutral categories. This analysis also helps in the recognition of feelings in Twitter data (tweets). This paper analyses sentiments shared through tweets during the COVID-19 pandemic, specifically for the healthcare sector. The purpose of this study is to examine people’s reactions toward the pandemic using different machine learning algorithms and to warn society about people’s mixed opinions. The tweets are extracted by considering three prompt areas of discussion on social media in COVID-19 situations. These three areas are the COVID-19 vaccine, post-COVID-19health factors and healthcare services.

### 1.1. Healthcare

#### 1.1.1. The COVID-19 Vaccine

The most debated topic on social media after the release of the COVID-19 vaccines were how they work and their consequences [9]. As per the details available from the Centers for Disease Control (CDC), various vaccines have been approved for COVID-19 [10,11]. Some of the approved vaccines are Pfizer BioNTech, Covid-shield, Covaxin and Moderna. Many people have tweeted their opinions about these vaccines [12]. Some discuss the pros and cons of vaccines after learning about their efficacy. People from different countries have different opinions about vaccination drives. In India, the first drive of COVID-19 vaccination started on 16 January 2021, with the Covid-shield and Covaxin vaccines.

#### 1.1.2. Post-COVID-19 Health Factors

People who had mild or severe symptoms of COVID-19 recovered quickly and with fewer complications [13], while patients with severe symptoms experienced a hard and long recovery, with most experiencing weakness, psychological and physiological disorders and fatigue [14]. As per a telephone survey conducted by the World Health Organization (WHO), around 20% of the people in the age group between 18 and 34 have protracted symptoms [15].

#### 1.1.3. Healthcare Service Providers

Healthcare service providers and workers, especially providers in direct contact with COVID-19 patients, have played a leading role in the pandemic and put their lives at risk, [16]. Sometimes, the front-line fighters deal with a variety of public assaults as well as physiological problems. They also must often cope with a lack of resources. People were expressing their opinions about the healthcare service providers during this crisis, and they were also utilizing social media to motivate frontline healthcare staff.

### 1.2. Contribution

The key contribution of this work is to

(1) Extract real-time Twitter data from healthcare sub-domains (COVID-19 vaccine, post-COVID-19 health factors and healthcare service providers) and use machine learning techniques to analyse and identify social media users’ narratives.

(2) Classification of users’ perceptions in the COVID-19 pandemic concerning three healthcare sub-domains: COVID-19 vaccine, post-COVID-19 health factors, and healthcare service providers with ML techniques. The purpose of this study is to understand how people are reacting to these queries and generate inferences.

## 2. Literature Survey

Diversified information is available in the literature based on the type of event and goal of the study. Sentiment analysis and effective computing is a field that helps in collecting public perceptions on political activities, commercial efforts, health crises and a wide range of other social events using social media platforms [17]. The continuous advancement in the field of artificial intelligence (AI) is the key to effective computing and sentiment analysis and has potential to analyze sentiments through huge data sets [18]. COVID-19 pandemic Twitter data analysis and swine flu Twitter data analysis were presented in articles [19] and [20], respectively.

A total of 242,525 healthcare tweets were collected from five Saudi Arabian areas and analyzed using K nearest neighbor, support vector machine (SVM) and naive Bayes algorithms [21]. The study [22] presented an analysis of 1,400,000 tweets by utilizing TF-IDF based correlation and latent Dirichlet allocation to identify key features and explore the interesting conclusions. A classification model based on clustering, TClustVID is presented in [23], in which data was drawn from the IEEE data repository. ML algorithms, such as SVM, Poisson, negative binomial and naive Bayes models are used to determine political opinions [24].

Many researchers presented different DL algorithms such as convolutional neural networks (CNN, recurrent neural networks (RNN) and long short-term memory (LSTM) [25,26] for analyzing different data. COVID-19 spread analysis using Twitter data considering travel history, age, gender and type of communication is presented in [27,28]. Table 1 shows some noteworthy contributions related to COVID-19 pandemic sentiment analysis. The table describes the data collection strategy, purpose, methods used, results and limitations. After the extensive literature survey, it was noted that a lot of research has been conducted on Twitter data analysis by applying different ML algorithms, even for the healthcare sector, but these works presented Twitter data analysis by considering generalized keywords such as COVID-19, corona virus, lockdown, health, etc. However, different sub-domains of the healthcare sector during the COVID-19 pandemic still need attention.

Existing work related to sentiment analysis as presented in Table 1 shows that the Twitter data were collected through common keywords during the COVID-19 pandemic. However, the analysis by considering a specific domain such as the healthcare domain, which this article is emphasizing, was untouched until now. Our objective is to analyze people’s sentiments for the healthcare domain and for specific queries such as ‘COVID-19 vaccine’, ‘post-Covid health factors’ and ‘Healthcare service providers’.

## 3. Data and Methods

**Figure 1:**
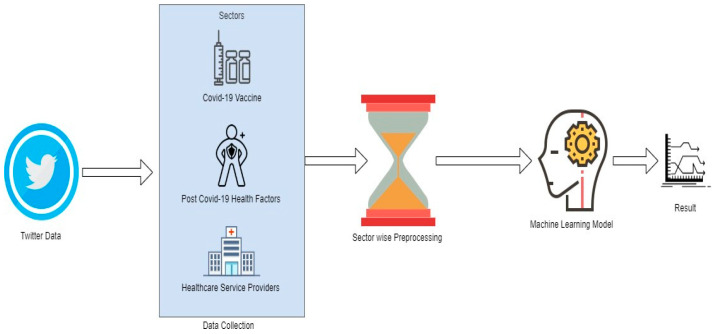
Processing structure.

The methodology in the paper starts with collecting related tweets and extends to pre-processing for each considered query, which includes data cleaning, transformation and applying ML techniques for sentiment analysis and classification to infer the people’s opinions, as shown in Figure 1. Classification techniques used are SVM, logistic regression (LR), multinomial naive Bayes (MNB), random forest (RF) and long short-term memory (LSTM). These multiple techniques will help in selecting the best fit model based on various performance matrices [36,37].

### 3.1. Data Collection

Data collection was conducted using the Twitter API, which requires the creation of a Twitter developer account in order to extract tweets. Token keys, API keys and secret keys are generated by a developer account so that the authorization procedure may be completed for collecting real time tweets. The Twitter data was retrieved using the Python library tweepy. The data was extracted for terms related to people’s opinions on the healthcare domain in COVID-19 scenarios, with a focus on vaccines, post-Covid health factors and healthcare service providers. The searched sample tweets are shown in Table 2. The number of tweets considered for each query was 10,000.

Table 3 shows the date range for each query. While collecting the tweets, we set the criteria that at least one tweet should be longer than 50 characters and in the English language only.

### 3.2. Sectorwise Preprocessing

**Figure 2:**
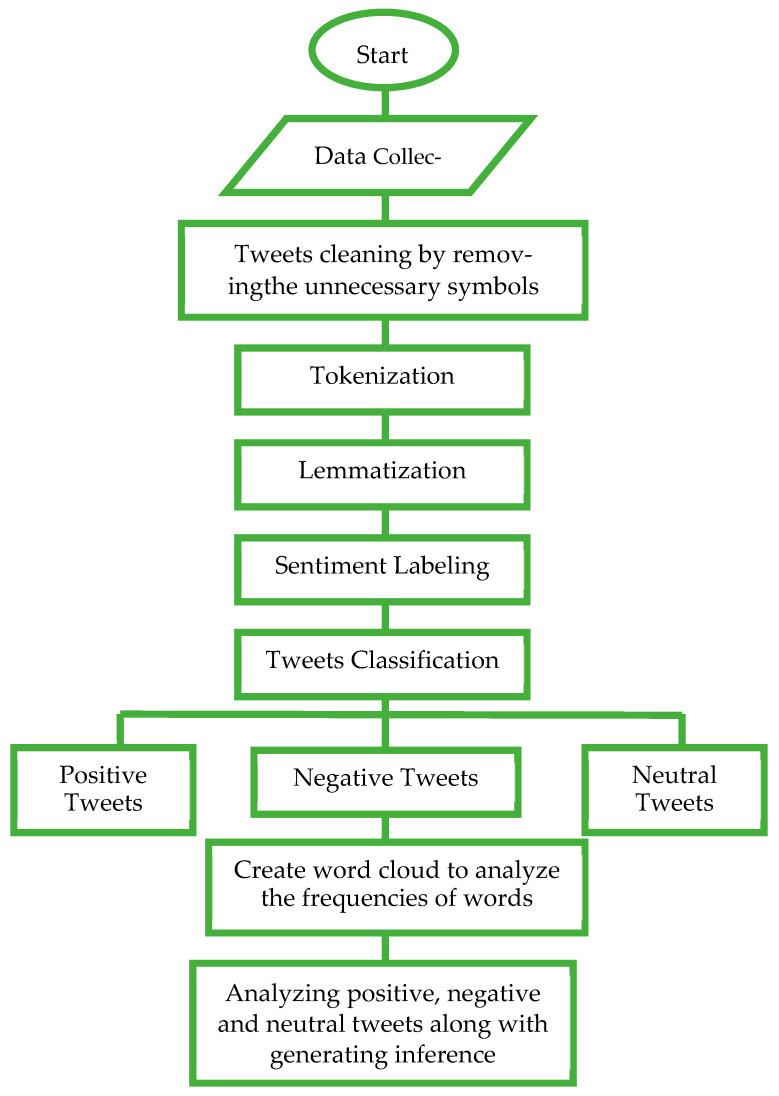
Flowchart: Preprocessing and sentiment analysis.

This section describes the preparation of the data as required by the ML models as seen in Figure 2. Tokenizing the tweets into a series of words, phrases or paragraphs was the first step. The next step was lemmatization, by which the root words were derived, which helped in finding the real meaning of words that have been used in tweets. Especially with social media data, it is essential to take care of the exact meaning of sentiments while calculating and vectorizing the sentiment values.

#### 3.2.1. Sentiment Calculation

The transformation of social media textual information into numerical information involves a calculative approach. Determining the attitude or the emotion of users, whether it is positive, negative or neutral requires a mathematical calculation. The Text-Blob package was used in the process of extracting the numerical sentiment values, which are float types and range from [−1, 1]. Text-blob, Vedar and Flair are three sentiment analyzers and perform wellin terms of accuracy of sentiment calculation. Text-blob was selected here for its good speed and accuracy [38].

#### 3.2.2. Sentiment Polarity Classification

After sentiment value calculations, the next step was to classify tweets into the categories shown in the pseudo code. The value provided by the Text-Blob package was used to determine the polarity classification. Here, we used three sentiment classifications, which included negative, neutral and positive. Each tweet’s polarity was determined by a score, which implies that if the score is greater than zero, the polarity categorization is positive; if it is equal to zero, it is neutral, and if it is less than zero, it is negative.

Pseudo code for Polarity Classification
*Start**Loop from i = 1 to number of tweets*
   *Calculate tweet Text polarity using TextBlob*

   *If polarity is greater than zero*

      *Tweet Text Sentiment = positive*

   *Else if polarity is less than zero*

      *Tweet Text Sentiment[i] = negative*

   *Else*

      *Tweet Text Sentiment[i] = neutral*
*End*

### 3.3. Machine Learning Models

#### 3.3.1. Support Vector Machine (SVM)

SVM is a vector-based learning system that is administered. In order to classify the data, vectors are drawn on the space. Hyper planes are used to draw conclusions and classify the data points by keeping the various classes of the data as far apart as possible. The machine is trained and hyper planes are generated using labelled data points. When completely new datasets are given, the machine quickly segregates them into one of the available classes. SVMs are applied in practice using a kernel. The capability to comprehend the hyper plane is achieved through linear algebra. It employs the inner product of supplied data instead of the observations themselves. Obtaining the sum of the products of each pair of input values yields the inner product. For example, the inner product of input vectors (a, b) and (c, d) is a*c + b*d, where a, b, c and dare symbolic vectors representations. The dot product of input and support vector, which is derived using the subsequent equation, is used to anticipate the inputs:

(1) $$f\left(x\right)=B_{0}+sum\left(a_{i}*\left(x,\;x_{i}\right)\right)$$

The input data’s inner product is computed using all of the data’s support vectors, and the coefficients of $B_{0}$ and $a_{i}\;$(for input values) must be determined using a learning technique while training. SVMs are less likely to over-fit and generalize better than other classifiers with more ability to fit the training data. SVM has many text classification applications and is suitable for Twitter data analysis.

#### 3.3.2. Logistic Regression (LR)

Logistic regression is a supervised classification approach that categorizes individuals into groups using a logistic function. For a given collection of features (or inputs), X; the target variable (or output), Y can only take discrete values. Logistic regression, contrary to popular assumption, is a regression model. The model creates a regression model to forecast the likelihood that a given data entry belongs to the “1” category. The sigmoid function is commonly employed as a logistic function, since it has the property of rising swiftly and exceeding the carrying capacity of the environment. The LR model detects a vector of variables in text classification and then calculates the coefficients for each input variable. The probability scale is constantly between 0 (never happens) and 1 (happens). In the case of binary classification, the likelihood of testing positive and not testing positive will equal 1. In logistic regression, the logistic function or sigmoid function is used to calculate probability. The logistic function is a basic S-shaped curve that converts input into a number between 0 and 1.

(2) $$h\theta \left(x\right)=1/\left(1+e^{-\left(\beta_{0}+\beta 1\;x\right)}\right)$$

hθ(x) is the output, where 0≤ hθ(x)≤1, β1 is the slope, $\beta_{0}$ is the y intercept, and *x* is the independent variable.

#### 3.3.3. Random Forest (RF)

Random forest is a technique for machine learning that can be used to solve problems involving regression and classification. It implements ensemble learning, a technique for solving complex problems by combining several classification algorithms. The random forest approach is made up of many decision trees. Bagging and/or bootstrapping clustering are used to train the random forest strategy’s ‘forest’. Bagging is a meta-algorithm that enhances the efficiency of machine learning algorithms by combining them. The outcome is determined by the random forest classifier, which is dependent on the decision tree forecasting. It makes forecasts by averaging the output of different trees. As the number of trees increases, so does the accuracy of the outcome. The disadvantage of the decision tree algorithms is overcome by the random forest method. It reduces dataset over-fitting and improves precision. The random forest method is capable of accurately classifying massive volumes of data and is suitable for tweet classification.

#### 3.3.4. Multinomial Naïve Bayes (MNB)

The multinomial naïve Bayes procedure is a popular probabilistic learning method in the area of natural language processing (NLP). The naïve Bayes classifier is composed of several algorithms, all of which share one point in common: each feature is classified unrelated to any other feature. The presence or absence of one feature has no direct effect on the absence or presence of any other feature. The naïve Bayes classifier is a group of probabilistic schemes that are based on the Bayes’ principle and the naïve concept of conditional independence across each pair of features and is able to predict tags from a text, which may be an email, any news or a tweet. The tagging of text is performedon a probability basis from a sample; the tag with the highest probability is selected. It considers a feature vector, which stores the frequency of appearance of a term. The probability P(c|x) is calculated by the Bayes theorem in which the probable outcomes class is *c*, and *x* is the delivered case to be recognized, which represents some specific characteristics.

(3) P(c|x)=P(x|c)* P(c)/P(x)

Naïve Bayes predicts a text’s tag. It calculates search tag’s probability for a given text and outputs the tag with the highest probability.

#### 3.3.5. Long Short-Term Memory (LSTM)

Recurrent neural networks (RNN) are one of the most widely used algorithms for sequential data, and they learn from past experience. The major problem with RNN is the long-term dependency, also known as the “vanishing gradient problem”, which means it does not perform well when working with vast amounts of data. LSTM has been introduced for taking care of long dependency problems, which helps in dealing with long textual data very accurately. This is the reason LSTM could be the best model to analyze social media data effectively. It is a particular category of recurrent neural network that is capable of learning long-term data relationships. The model’s recurring module makes it possible because it has up to four interconnected layers. An LSTM module consists of a cell state and three gates, allowing it to selectively comprehend, unlearn or recall information from each unit. In LSTM, the cell state allows information to pass through units without being affected by some linear interactions. Each unit has an input, an output and a forget gate, which adds or subtracts data from the cell state. A sigmoid function is employed by the forget gate to evaluate whether the information from the earlier cell state should be overlooked. To regulate the information flow into the latest cell state, the input gate performs a point-wise multiplication of ‘sigmoid’ and ‘tanh’. Finally, the gate in output decides which data to transfer to the next concealed state.

#### 3.3.6. Experimental Work

Python3 Libraries such asnumpy, scipy, scikit-learn, keras, pandas, nltk, tweepy, matplotlib, etc. were used to build a predictive model and sentiment identification using ML models. The parameters used for different models are:

SVM:

loss function = ‘squared_hinge’

max_iteration = 1000

LR:

solver = ‘lbfgs’,

max_iter = 1000

RF:

n_estimators = 100,

criterion = ‘gini’

MNB:

alpha (smoothing parameter) = 1.0

LSTM:

Loss = ‘categorical_crossentropy’,

Optimizer = ‘adam’

activation function = ‘softmax’

The remaining parameters for all models were kept on their default values.

### 3.4. Performance Evaluation Matrix

Classifier accuracy is measured with the help of a confusion matrix. This matrix provides the number of right and wrong predictions by comparing actual target values. It consists of four parameters: (1) true positive (TP): shows how many actual true values the model predicted as true; (2) true negative (TN): shows how many actual false values the model predicted as false; (3) false positive (FP): shows how many actual false values are predicted as true; and (4) false negative (FN): shows how many actual true values are predicted as false.

#### 3.4.1. Accuracy

Accuracy shows the percentage of right predictions. The formula for accuracy calculation is presented in Equation (4),

(4) $$\mathrm{Accuracy}=\frac{\mathrm{TP}+\mathrm{TN}}{\mathrm{TP}+\mathrm{TN}+\mathrm{FP}+\mathrm{FN}}$$

#### 3.4.2. Precision

This attribute shows how regularly a model predicts a true positive. Low values for this parameter show large numbers of false positives. The formula for computing precision is presented in Equation (5),

(5) $$\mathrm{Precision}=\frac{\mathrm{TP}}{\mathrm{TP}+\mathrm{FP}}$$

#### 3.4.3. Recall

Accuracy does not provide the information about FP and FN. In some cases, values of FN and FP are substantial. Recall and F1 score show a very significant role in these cases. Recall shows information regarding false negative predictions. Lower values of the recall parameter mean a large number of false negatives. The formula for Recall is presented in Equation (6),

(6) $$\mathrm{Recall}=\frac{\mathrm{TP}}{\mathrm{TP}+\mathrm{FN}}$$

#### 3.4.4. F1 Score

This parameter is obtained by combining recall and precision. A high value of recall shows a low number of false negatives and false positives. Equation (7) present formulas for the F1 score,

(7) $$F1\;\mathrm{score}=2*\frac{\mathrm{Precision}*\mathrm{Recall}}{\mathrm{Precision}+\mathrm{Recall}}$$

## 4. Results

### 4.1. Word Cloud Visualization

**Figure 3:**
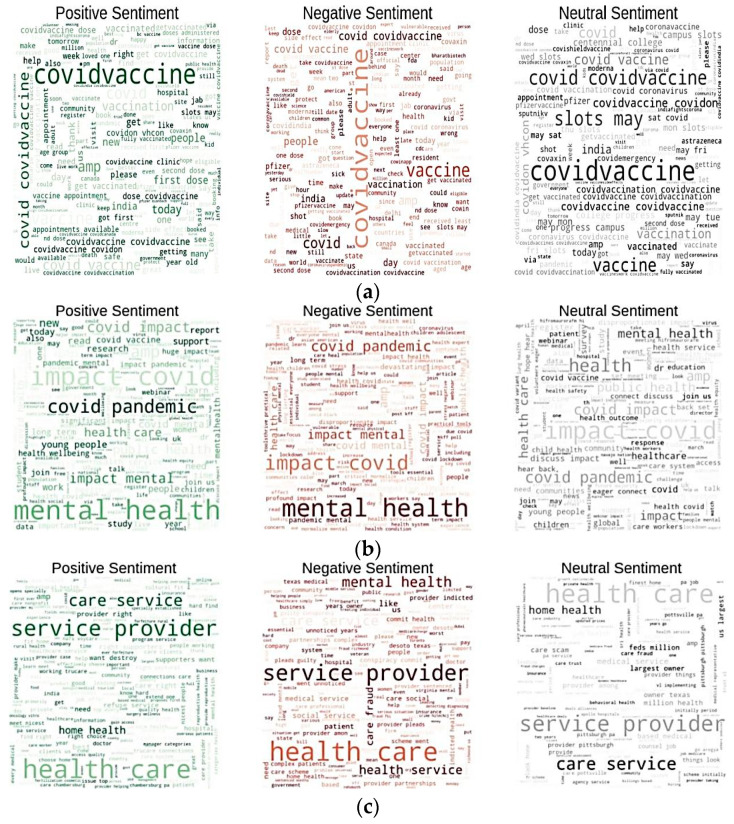
Word clouds for: (**a**) COVID-19 vaccine; (**b**) COVID-19 health factors; (**c**) health service providers.

Visualization provides a deeper understanding of data and the types of information that may be extracted from them. We depicted the word clouds using the Python3 NLTK library to investigate the various types of terms tweeted by users and visualize the frequently occurring words for all considered domains. Figure 3a–c show word clouds visualization for COVID-19 vaccine, post-Covid health factors and healthcare service providers, respectively. Each figure contains word clouds for three sentiments which are positive, negative and neutral.

Figure 3a shows that the most frequently used words for the ‘COVID-19 vaccine’ query for positive sentiments were ‘dose’, ‘first’, ‘available’, ‘appointment’ ‘slots’, ‘clinic’, etc.; frequently used words for negative sentiments were ‘world’, ‘day’, ‘dose’, ‘India’, ‘still’, ‘kids’, ‘vaccine’, ‘people’, etc.; and frequently used words for neutral sentiments were ‘slots’, ‘Covid’, ‘may’, ‘vaccinated’, etc. The words ‘please’, ‘first’, right were available only in the positive sentiment word cloud, while the words ‘wrong’, sick, death were present only in the negative sentiment word cloud.

Figure 3b shows the that most frequently used words for ‘the post-Covid health factors’ query for positive sentiments were ‘Covid pandemic’, ‘support’, ‘metal health’, ‘impact’, ‘report’, ‘young’, ‘people’, etc.; frequently used words for negative sentiments were ‘mental health’, ‘long-term’, ‘impact’, ‘student’, ‘research’, ‘Covid lockdown’, etc.; and frequently used words for neutral sentiments were ‘health’, ‘education’, ‘mental health’, ‘health outcome’, ‘discuss’, ‘healthcare’, ‘impact’, etc. The words ‘study’, ‘school’, ‘work’, ‘join’ were present only in the word cloud of positive sentiments, while the word ‘long-term’ was present only in the word cloud of negative sentiment.

Figure 3c shows that the most frequently used words for ‘healthcare service provider’ query for positive sentiments were ‘care’, ‘service’, ‘home’, ‘need’, ‘destroy’, ‘provider’, ‘right’, etc.; for negative sentiments were ‘mental health’, ‘patient’, ‘fraud’, ‘medical taxes’, ‘care’, ‘government’, etc.; and for neutral sentiments were ‘health outcome’, ‘response’, ‘healthcare’, ‘service’, ‘home’, ‘largest owner’, ‘feeds’, ‘million’, etc. The words ‘right’ and ‘provider’ were present only in the word cloud of positive sentiments, while the word ‘medical taxes’ was present only in the word cloud of negative sentiment.

### 4.2. Tweets Polarity and Percentage

**Figure 4:**
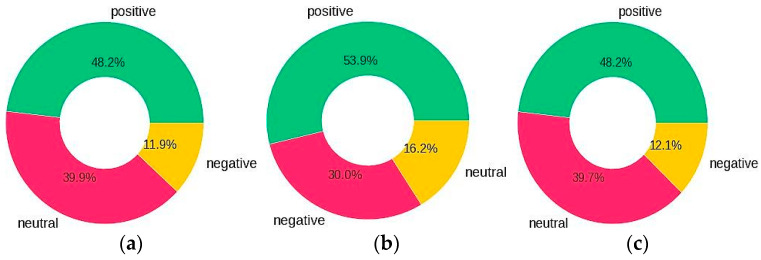
Percentage of tweets for: (**a**) COVID-19 vaccine; (**b**) post-Covid health factors; (**c**) health service providers.

Figure 4a–c show the percentage of polarity of tweets after sentiment labelling for all domains for the COVID-19 vaccine, post-Covid health factors and healthcare service provider queries, respectively. By exploring the sentiments for all queries, the results show that for the ‘COVID-19 vaccine’ query, 48.2%, 11.9% and 39.9% of the tweets people shared were classified as positive, negative and neutral sentiments, respectively. For the ‘post-Covid health factors’ query, 48.2%, 12.1% and 39.7% of the tweets people shared were classified as positive, negative and neutral sentiments, respectively. For ‘health care service providers’ query, 53.8%, 16.2% and 30.0% were positive, negative and neutral sentiments, respectively.

The results present assessment with the employed five ML models: SVM, LR, RF, MNB and LSTM. We divided the collected data into training (75% of data) and test sets (25% of data) to evaluate the models. The outcomes of all algorithms considered were evaluated using F1 score, precision, recall and accuracy (for the test sets). Table 4, Table 5, Table 6, Table 7 and Table 8 show the value of these parameters for SVM, LR, RF, MNB and LSTM, respectively.

## 5. Discussion

The goal of this work is to extract tweets from healthcare sub-domains around the world and assess sentiments during the COVID-19 pandemic. COVID-19 has had a devastating impact on individuals, both directly and indirectly. Affected individuals have shared their health related experiences on social media platforms. A flood of data is continuously being produced as a result of COVID-19’s continued effect. Analyzing this enormous volume of data using conventional statistical methods is difficult.

**Figure 5:**
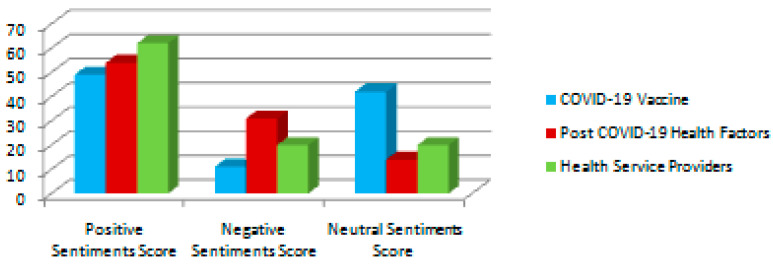
Sentiment comparison for all queries.

Figure 5 shows the comparison of the sentiment percentage of each query. It depicts that the most positive sentiments were collected for the ‘healthcare service provider’ query, which leads to the inference that people are very happy with the healthcare service providers. They appreciate the efforts and hard work of doctors, nurses, hospital management and government support services. The query ‘Post-Covid health factor’ received the most negative sentiments, indicating that people experienced numerous negative post-Covid issues.

**Figure 6:**
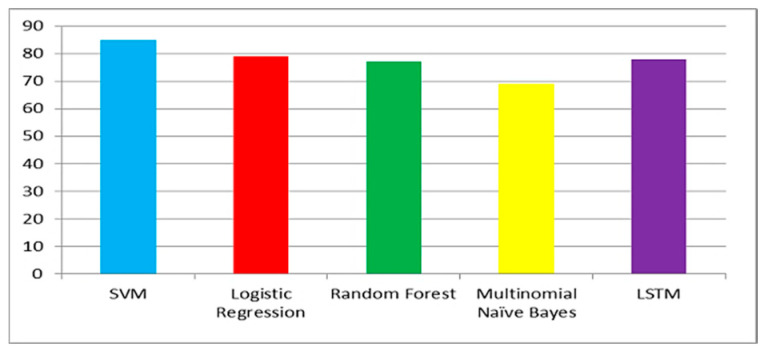
Model accuracy comparison for the COVID-19 vaccine query.

**Figure 7:**
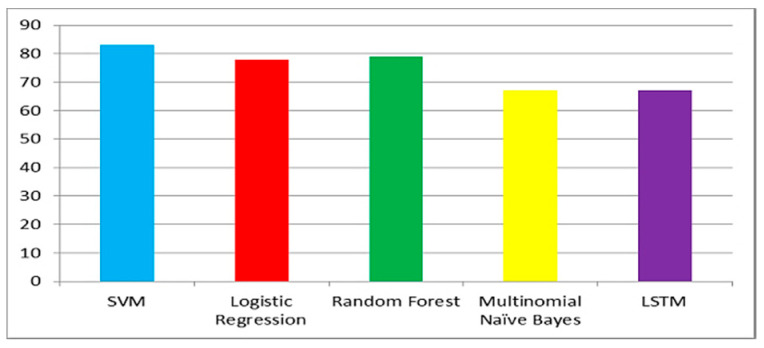
Model accuracy comparison for the post-COVID-19 health factors query.

**Figure 8:**
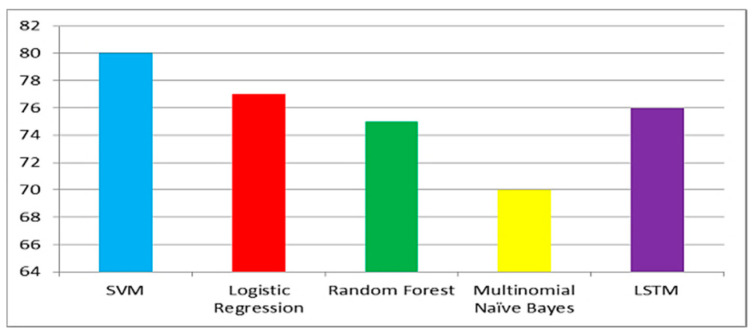
Model accuracy comparison for the healthcare service provider query.

Figure 6, Figure 7 and Figure 8 show the accuracy comparison of all considered ML models for each query. Prediction accuracy values calculated by SVM, LR, RF, MNB and LSTM for ‘COVID-19 vaccine’ query are 85%, 79%, 77%, 69% and 78%, respectively. Prediction accuracy values calculated by SVM, LR, RF, MNB and LSTM for ‘post-Covid health factors’ query are 83%, 78%, 79%, 67% and 71%, respectively. Prediction accuracy values calculated by SVM, LR, RF, MNB and LSTM for ‘healthcare service providers’ query are 80%, 77%, 75%, 70% and 76% respectively. These results indicate that SVM provided the highest accuracy for all queries when compared with the other considered models.

Different kinds of performance metrics give us a clear idea of how to deal with large and imbalanced datasets. It is very difficult to manage Twitter data because of the imbalanced ratio of positive, negative and neutral tweets. Apart from accuracy, we tested the F1 score as well and found that for the ‘COVID-19 vaccine’ query, the highest scores for negative sentiments, neutral sentiments and positive sentiments were 0.54, 0.88 and 0.89, respectively, and they were achieved through SVM. The maximum F1 score obtained for positive sentiments was 0.89.The highest values of F1 scores for the ‘post-Covid health factors’ query for negative sentiments, neutral sentiments and positive sentiments were 0.8, 0.74 and 0.87, respectively, and were achieved through SVM. For positive sentiments, the maximum F1 score obtained was 0.87.The highest values of F1 scores for the ‘healthcare service provider’ query for negative sentiments, neutral sentiments and positive sentiments were 0.61, 0.87 and 0.74, respectively, and were achieved through SVM. For positive sentiments, the maximum F1 score obtained was 0.87.

In all queries, the maximum F1 score was achieved through SVM for positive sentiments, which implies that there are very few false positives and false negatives predicted by SVM for positive sentiments. In future, more Tweets can be extracted to assess the impact of pandemics in other domains such as, education, economy, food industry, tours and travel industry and disaster management. The novel machine learning approaches such asmeta-heuristic-algorithm-based tuning; the ensemble approach and hybrid modes can be utilized for sentiment predictions in different domains.

## 6. Conclusions

Social media platforms serve as a forum for people to express themselves and their ideas. Policymakers may benefit from an examination of these ideas. The novelty of this work is the sentiment analysis on the basis of a specific domain query, which is healthcare. For the case of the COVID-19 pandemic, we selected Twitter data from three sub-domains of healthcare: COVID-19 vaccine, post-Covid health factors and healthcare service providers, for analysis. Analysis was conducted through sentiment visualization and ML procedures.

The practical application of sentiment analysis lies in the decision solutions to handle the pandemic situations more effectively. The correct analysis of sentiments will help in protecting emotional health of people during the pandemic.

Sentiment visualization is offered using the aid of word clouds, pi-charts and bar charts. These visualization tools depict that maximum positive sentiments were collected for the query ‘healthcare service provider’, while the maximum negative sentiments were collected for the query ‘post-Covid health factors’. The ML algorithms considered in this work are SVM, LR, RF, MNB and LSTM. The accuracy, precision, recall and F1 score of all machine learning algorithms were determined. SVM achieved the maximum F1 score, precision, recall and accuracy, which were 0.89 (for positive sentiment tweets), 0.92 (for neutral sentiment tweets) and 0.85 (for negative sentiment tweets), respectively, for the COVID-19 vaccine query.

## Figures and Tables

**Table 1:** Summary of literature review related to COVID-19.

Paper (Ref. No.)	Twitter Data Collection Criteria/Keywords	Purpose (Related to COVID-19 Pandemic)	Methods Used	Results	Limits/Issues
[29]	#IndiaLockdown, #IndiafightsCorona	People sentiments for lockdown	Statistical methods	People’s attitude for lockdown	Accuracies are low.
[30]	Sentiment data during Covidpeak.	Sentiment analysis of US peoples.	Naïve Bayes (NB)&logistic regression (LR).	Accuracies are 91% (NB) and 74%(LR).	Short tweets are used.
[31]	Semantic comments on reddit	Identification of peoples/patients sentiments.	LSTM& RNN based NLP	Accuracy is only 81.15%	Accuracy is fairly low.
[32]	#COVID-19, #coronavirus	People’s response and feelings	Naïve Bayes (NB) model	Reactions polarities.	Accuracy not shown
[33]	COVID-19 data	Public attention trends	K-means using min–max normalization	Three-level n-grams tweets analysis	Accuracy not shown
[34]	Data of COVID-19 vaccination	People’s opinions and sentiments	Valence Aware Dictionary and Sentiment Reasoner (VADER)	People’s different sentiments	Performance accuracy not shown
[35]	Related keywords	For analysing the symptoms	Natural language processing (NLP) model	Results for symptoms, fever/pyrexia (66.1%), etc.	Accuracy not shown

**Table 2:** Collected tweets sample.

Query	Sample Tweets	Date
COVID-19 vaccine	BBC news saying, vaccines only 33 effective against Indian variant until 2nd jab when nearer 90 COVID-19 Vaccine	23 May 2021
	I’m done with both doses against COVID-19 Vaccine	23 May 2021
Post-Covid health factors	Study Healthy young adults who had COVID-19 may have long term impact on blood vessels amp heart health	23 May 2021
	What about those who are facing death in their family COVID-19 is not only harming the respiratory system but it is having a huge impact on mental health too cancel board exam cancel12thboardexam	23 May 2021
Healthcare service provider	See results from a randomized open label study of 5795 COVID-19 hospitalized patients received high titer convalescent plasma plus usual care in 177 UK National Health	23 May 2021
	Sofarmorethan250peoplehavereceivedfree health care and consultation via telemedicine service For any health related problems or COVID related consultation call us NOW Tollfree numbers 16605152003 NTC 9801573330Ncell	23 May 2021

**Table 3:** Collected twitter data details.

Query	StartDate	End Date	No. of Tweets
COVID-19 vaccine	10 May 2021	23 May 2021	11,113
Post-Covid health factors	5 December 2020	23 May 2021	10,061
Healthcare service provider	23 March 2020	25 May 2021	10,059

**Table 4:** SVM model evaluation results for all three considered queries.

Query	Polarity of Tweets	Precision	Recall	F1Score	Accuracy (in %)
COVID-19 vaccine	Negative	0.68	0.45	0.54	85
	Neutral	0.84	0.92	0.88	
	Positive	0.89	0.88	0.89	
Post-Covid health factors	Negative	0.81	0.79	0.8	83
	Neutral	0.73	0.76	0.74	
	Positive	0.87	0.87	0.87	
Healthcare service providers	Negative	0.66	0.56	0.61	80
	Neutral	0.75	0.73	0.74	
	Positive	0.85	0.89	0.87	

**Table 5:** Logistic regression model evaluation results for all three considered queries.

Query	Polarity of Tweets	Precision	Recall	F1Score	Accuracy (in %)
COVID-19 vaccine	Negative	0.43	0.46	0.44	79
	Neutral	0.83	0.82	0.82	
	Positive	0.86	0.85	0.85	
Post-Covid health factors	Negative	0.76	0.75	0.75	78
	Neutral	0.60	0.67	0.64	
	Positive	0.86	0.84	0.85	
Healthcare service providers	Negative	0.54	0.53	0.53	77
	Neutral	0.69	0.70	0.69	
	Positive	0.85	0.85	0.85	

**Table 6:** Random forest model evaluation results for all three considered queries.

Query	Polarity of Tweets	Precision	Recall	F1Score	Accuracy (in %)
COVID-19 vaccine	Negative	0.93	0.20	0.33	77
	Neutral	0.78	0.82	0.80	
	Positive	0.76	0.87	0.81	
Post-Covid health factors	Negative	0.85	0.67	0.75	79
	Neutral	0.77	0.60	0.68	
	Positive	0.77	0.91	0.83	
Healthcare service providers	Negative	0.80	0.28	0.42	75
	Neutral	0.77	0.51	0.61	
	Positive	0.74	0.95	0.84	

**Table 7:** Multinomial naïve Bayes model evaluation results for all three considered queries.

Query	Polarity of Tweets	Precision	Recall	F1Score	Accuracy (in %)
COVID-19 vaccine	Negative	0.86	0.04	0.08	69
	Neutral	0.81	0.59	0.68	
	Positive	0.64	0.82	0.75	
Post-Covid health factors	Negative	0.82	0.39	0.53	67
	Neutral	0.77	0.25	0.37	
	Positive	0.64	0.95	0.77	
Healthcare service providers	Negative	0.84	0.14	0.24	70
	Neutral	0.83	0.27	0.41	
	Positive	0.69	0.98	0.81	

**Table 8:** LSTM model evaluation results for all three considered queries.

Query	Polarity of Tweets	Precision	Recall	F1Score	Accuracy (in %)
COVID-19 vaccine	Negative	0.46	0.25	0.32	78
	Neutral	0.76	0.87	0.81	
	Positive	0.84	0.84	0.84	
Post-Covid health factors	Negative	0.68	0.63	0.65	67
	Neutral	0.59	0.48	0.53	
	Positive	0.76	0.84	0.8	
HealthCare service providers	Negative	0.55	0.57	0.56	76
	Neutral	0.60	0.27	0.37	
	Positive	0.84	0.91	0.87	

## Data Availability

Not applicable.
